# Toxicity of orally administrated biodegradable mesoporous silica nanoparticles in mice

**DOI:** 10.3389/fbioe.2026.1834001

**Published:** 2026-06-30

**Authors:** Jie Li, Xin Zhao, Zhen Wang, Xiaochun Xie, Wanfen Li, Yingshi Han, Xin Tang, Jiayi Wang, Yige Shan, Fan Zhang, Yingshuai Wang, Yi He

**Affiliations:** 1 Department of Neurology, The Third Affiliated Hospital of Southern Medical University, Guangzhou, Guangdong, China; 2 School of Medicine, South China University of Technology, Guangzhou, Guangdong, China; 3 Department of Breast Surgery, Second Hospital of Jilin University, Changchun, China; 4 Department of Rheumatology and Immunology, The Third Affiliated Hospital of Southern Medical University, Guangzhou, Guangdong, China; 5 The First Affiliated Hospital, Biomedical Translational Research Institute and School of Pharmacy, Jinan University, Guangzhou, China; 6 School of Pharmacy, Xuzhou Medical University, Xuzhou, China; 7 State Key Laboratory of Advanced Medical Materials and Devices, Institute of Biomedical Engineering, Tianjin Institutes of Health Science, Chinese Academy of Medical Science & Peking Union Medical College, Tianjin, China; 8 Intensive Care Unit, Affiliated Hospital of Shandong Second Medical University, Weifang, Shandong, China

**Keywords:** degradation, mesoporous silica nanoparticles, oral administration, organic-inorganic hybrid, toxicity

## Abstract

Organic–inorganic hybrid mesoporous silica nanoparticles (MSNs) have emerged as promising platforms in biomedicine owing to their architecturally sophisticated frameworks, stimulus-responsive properties, and tunable functionalities. Despite their potential, the influence of framework composition on the *in vivo* fate of MSNs remains insufficiently characterized. Here, we systematically evaluate the toxicity, biodistribution, and immunological impact of MSNs featuring three distinct frameworks—pure Si-O, disulfide-bridged, and diselenide-bridged matrices—following oral administration in mice. None of the MSN variants induced mortality at doses up to 2000 mg/kg. Comprehensive hematological, biochemical, and histopathological analyses revealed no overt toxicity upon repeated exposure. Notably, diselenide-bridged MSNs, characterized by enhanced degradability, exhibited accelerated clearance from the liver, spleen, and kidneys relative to their counterparts. Furthermore, all MSN types demonstrated a high degree of immunological safety, as evidenced by unaltered immune cell profiles and the absence of significant inflammatory responses. These findings provide mechanistic insights into the design of organic–inorganic hybrid MSNs with controllable degradability and clearance kinetics, informing their rational development for biomedical applications.

## 1 Introduction

Silicon, a prevalent trace element in the human body, underpins critical physiological functions, with its oxidized form, silica, exhibiting bioavailability and diverse biological activities (Jugdaohsingh et al., 2002; Nielsen, 2014; Fu et al., 2022; Lu et al., 2026). When silicon atoms react with oxygen, the resulting silica becomes bioavailable and can exhibit biological activities (Croissant et al., 2018; Dogra et al., 2018; Huang et al., 2021; Ji et al., 2019; Florek et al., 2017). Classified as Generally Recognized as Safe” (GRAS) by the FDA (ID Code: 14808-60-7), silica-based materials are widely employed in food additives, pharmaceuticals, and diagnostic probes (Yang et al., 2020). Notably, silica nanoparticles, such as Cornell dots, have gained FDA approval for molecular imaging in brain tumor diagnostics, highlighting their translational potential in oncology (Phillips et al., 2014). The advent of mesoporous silica nanoparticles (MSNs) has further expanded their utility, leveraging large surface areas, tunable pore architectures (Tang et al., 2012; Li and Zhou, 2023; Liang et al., 2025; He et al., 2024), and versatile surface functionalization for targeted drug delivery and controlled release in specific microenvironments (Li et al., 2022; Heidari et al., 2024; Romaní-Cubells et al., 2024; Li et al., 2019; Xu et al., 2023).

Despite these advances, toxicity concerns remain a significant barrier to the clinical adoption of MSNs (Abbasi et al., 2023; Younis et al., 2022; Kim et al., 2015). While silica is generally considered biocompatible, reports of adverse effects—such as hemostatic toxicity, cytotoxicity, immune activation, and potential cancer metastasis following intravenous or inhalational exposure—raise caution (Croissant et al., 2020; Huang et al., 2022; Hossen et al., 2018; Mohammadpour et al., 2019). Oral exposure, the primary route of silica intake through diet and pharmaceuticals, necessitates a deeper understanding of MSN safety profiles. Although silica degrades into non-toxic silicic acid in biological systems, incomplete degradation can lead to toxicity, influenced by physicochemical properties, dosage, exposure duration, and microenvironmental factors (Chen et al., 2025; Croissant et al., 2017). Recent developments in organic-inorganic hybrid MSNs have introduced tailored frameworks that combine the structural advantages of silica with stimulus-responsive functionalities (Peter et al., 2021; Lérida-Viso et al., 2023; Jafari et al., 2019; Wang et al., 2021). For instance, disulfide-bridged MSNs enable glutathione (GSH)-responsive degradation and drug release (Xiao et al., 2021), while diselenide-bridged MSNs offer enhanced biodegradability and responsiveness to GSH, reactive oxygen species (ROS), and X-ray radiation, with applications in tumor-targeted drug delivery and anti-inflammatory therapies (Yang et al., 2022; Shao et al., 2020). These hybrid frameworks confer distinct physicochemical and degradative properties compared to conventional Si-O-based MSNs, critically influencing their *in vivo* behavior (Shi et al., 2022; Peng et al., 2022). However, the toxicity and biodistribution of these hybrid MSNs, particularly via oral administration, remain underexplored.

**SCHEME 1:**
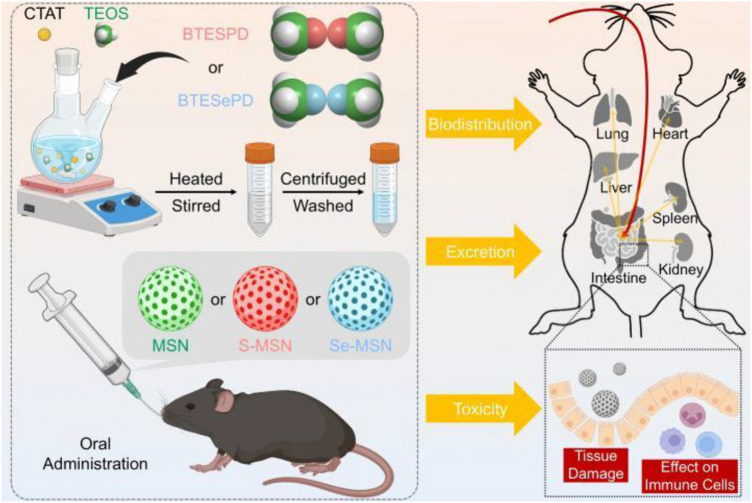
Systematic evaluation of organically bridged mesoporous silica nanoparticles (MSNs) after oral administration in mice demonstrates high biocompatibility and framework-dependent behavior in vivo. Although all MSN variants showed excellent immunological safety without observable toxicity, diselenide-bridged MSNs cleared more rapidly from major organs owing to their enhanced degradability. These results offer key design principles for developing organic-inorganic hybrid MSNs with controllable degradation kinetics for biomedical use.

Here, we report the synthesis and characterization of MSNs with three distinct frameworks: pure Si-O, disulfide-bridged, and diselenide-bridged matrices (Scheme 1). We systematically assessed their physicochemical properties, *in vitro* degradability, and *in vivo* toxicity, biodistribution, and immunological effects following oral administration in mice. Our findings elucidate the role of framework composition in modulating clearance kinetics and safety profiles, providing a foundation for the rational design of hybrid MSNs for biomedical and industrial applications.

## 2 Materials and methods

### 2.1 Synthesis and characterizations

Mesoporous silica nanoparticles (MSNs), including pure Si–O (MSN), disulfide-bridged (S-MSN), and diselenide-bridged (Se-MSN) variants, were synthesized following established protocols (Yang et al., 2022; Lu et al., 2023). Morphological features were characterized using a JEM-2100F transmission electron microscope (JEOL, Ltd., Japan). Hydrodynamic diameter and zeta potential were measured in aqueous solution with a Nano-ZS 90 Nanosizer (Malvern Instruments Ltd., UK). Specific surface area and pore size distributions were determined *via* Brunauer–Emmett–Teller (BET) and Barrett–Joyner–Halenda (BJH) methods, respectively. Sulfur and selenium contents in S-MSN and Se-MSN were quantified by inductively coupled plasma optical emission spectrometry (ICP-OES).

### 2.2 Anesthesia of mice

Mice requiring anesthesia were anesthetized with 2.5% isoflurane inhalation using an RWD520 anesthesia system (RWD Life Science Co., Ltd., China) connected to an air pump (Model R510-25) for continuous gas delivery. As blood sampling and target organ collection were performed immediately after anesthesia induction, no maintenance phase was required, and 2.5% isoflurane was used throughout the procedure.

### 2.3 Biodistribution of MSNs

To investigate the biodistribution of MSNs, 36 female C57BL/6 mice were randomly assigned to four groups receiving daily oral administration of MSNs (10 mg kg^-1^) or vehicle for 7 days. At 7, 28, and 90 days post-administration, mice were euthanized, and major organs (liver, spleen, lungs, heart, kidneys, intestine) were collected. Silicon content was quantified using inductively coupled plasma mass spectrometry (ICP-MS). Tissue samples were weighed, digested overnight in 1 mL of 65% nitric acid, treated with 3 mL of 30% hydrogen peroxide, and heated to form an ash residue. The residue was dissolved in 2% nitric acid for silicon analysis.

### 2.4 Mouse immune cell isolation and flow cytometry

To evaluate the immunological impact of MSNs, female C57BL/6 mice were randomized into four groups: wild-type (WT), MSNs with three distinct frameworks-pure Si-O (MSN), disulfide-bridged (S-MSN), and diselenide-bridged (Se-MSN). Following euthanasia, body weight and the weights of the liver and spleen were recorded. Immune cells were isolated from the liver, spleen, and intestinal tissues, and their phenotypic profiles were analyzed by flow cytometry using fluorochrome-conjugated antibodies: FITC-anti-Gr-1, PerCP-Cy5.5-anti-CD11c, APC-anti-CD44, Alexa Fluor 700-anti-MHC-II, APC-Cy7-anti-CD45, BV421-anti-CD3, BV510-anti-CD11b,BV605-anti-CD69,BV711-anti-CD8α,BV786-anti-Lag3,PE-CF594-anti-PD-1, PE-Cy5-anti-CD19, and PE-Cy7-anti-NK1.1. Cell suspensions were analyzed on an LSRFortessa flow cytometer (BD Biosciences), with data processed using FlowJo software (Tree Star, Inc.).

## 3 Results

### 3.1 Physicochemical characterization and degradation of MSNs

**FIGURE 1:**
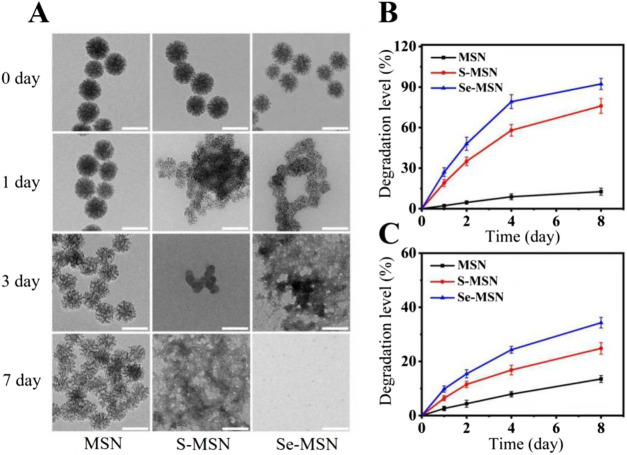
Degradation manner of three types MSNs. **(A)** TEM images of each MSNs under 10 mM GSH at different times (0, 1, 3, and 7 days), scale bars represent 50 nm. **(B,C)** Degradation level of each MSN was determined by the quantitative measurement of Si under **(B)** 10 mM or **(C)** 10 μM GSH-containing SBF.

MSNs with three distinct frameworks—pure Si-O (MSN), disulfide-bridged (S-MSN), and diselenide-bridged (Se-MSN)—were synthesized by varying the ratios of inorganosilane and organosilane precursors. Transmission electron microscopy (TEM) confirmed uniform particle diameters of 70 ± 10 nm across all MSN types (Figure 1A). Hydrodynamic diameters and negative surface charges were comparable (Supplementary Table S1). Brunauer–Emmett–Teller (BET) analysis revealed specific surface areas of 523.3, 492.6, and 553.8 m2 g^-1^ for MSN, S-MSN, and Se-MSN (Supplementary Table S1), respectively, with pore volumes of 1.24, 1.19, and 1.06 cm^3^ g^-1^, and average pore sizes of 5.8, 6.2, and 6.5 nm (Supplementary Table S1). Inductively coupled plasma optical emission spectrometry (ICP-OES) verified sulfur and selenium contents of 9.9% in S-MSN and Se-MSN, respectively. *In vitro* degradation studies were conducted in simulated biological fluids, including 10 mM and 10 μM GSH-containing SBF buffer, simulated gastric fluid (SGF), and simulated intestinal fluid (SIF) at 37 °C, to mimic intracellular, extracellular and gastrointestinal environments. In SGF, MSN, S-MSN, and Se-MSN showed minimal degradation within 8 h, with Se-MSN displaying the highest degradation level but still remaining below 4%. In SIF, all nanoparticles exhibited time-dependent degradation over 7 days, with Se-MSN degrading more rapidly than S-MSN and MSN. Under 10 mM GSH, S-MSN and Se-MSN exhibited rapid degradation, disintegrating into fragments by day 3 and fully degrading by day 7 (Supplementary Figure S1A). In contrast, pure Si–O MSN showed minimal degradation under identical conditions. Quantitative silicon release confirmed that Se-MSN degraded faster than S-MSN in both GSH concentrations (Figures 1B,C), likely due to the lower bond energy of diselenide (172 kJ mol^−1^) compared to disulfide (240 kJ mol^−1^) bonds, enhancing responsiveness to reductive environments. Cytotoxicity assays (SRB) in HUVEC, RAW 264.7 and HIEC-6 cells revealed dose-dependent toxicity, with no significant effects below 100 μg/mL (Supplementary Figure S2).

### 3.2 Acute oral toxicity

The acute toxicity of the three MSN variants was evaluated in female mice at oral doses of 1000 and 2000 mg/kg for over 14 days. Daily monitoring revealed no clinical signs of toxicity, abnormal behavior, or changes in body weight. No mortality, clinical signs of toxicity, abnormal behavior, or body weight changes were observed. Gross and histopathological examinations of major organs (heart, lung, liver, spleen, kidney, colon) showed no abnormalities (Supplementary Figure S3), indicating a maximum tolerated dose (MTD) exceeding 2000 mg kg^-1^, consistent with prior reports of low acute toxicity for MSNs in rats.

### 3.3 Repeated-dose toxicity

**FIGURE 2:**
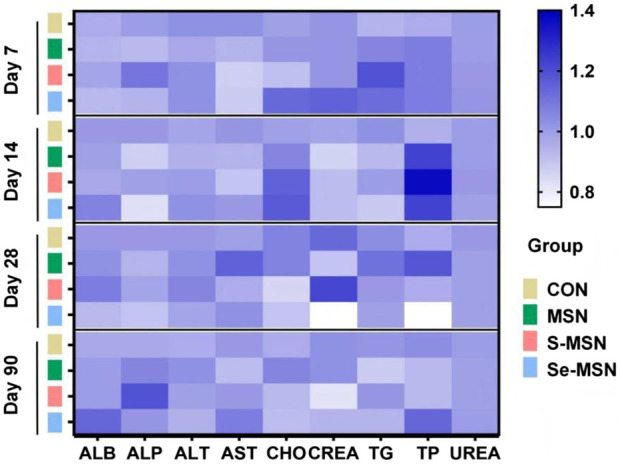
The blood biochemistry data of mice after repeated challenging of three types of MSNs. Mice were treated with each MSN at 10 mg/kg daily for 1 week. Then, they were sacrificed on day 7, 14, 28 and 90 after the first challenging. Albumin (ALB), alkaline phosphatase (ALP), alanine aminotransferase (ALT), aspartate aminotransferase (AST), total cholesterol (CHO), serum creatinine (CREA), total triglycerides (TG), total protein (TP), and serum urea (UREA), were selected for analysis. Each bar represents the mean ± SD, n = 6. P* < 0.05.

**FIGURE 3:**
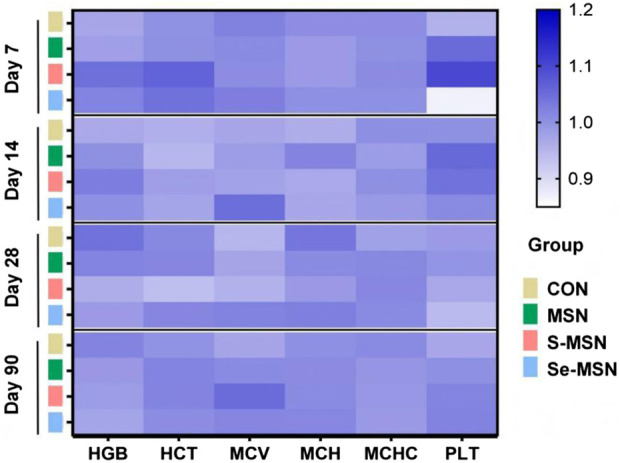
The hematological factors of mice after repeated challenging of three types of MSNs. Mice were treated with each MSN at 10 mg/kg daily for 1 week. Then, they were sacrificed on day 7, 14, 28 and 90 after the first challenging. Hemoglobin level (HGB), hematocrit value (HCT), mean corpuscular volume (MCV), mean corpuscular hemoglobin (MCH), mean corpuscular hemoglobin concentration (MCHC), and platelet count (PLT) were selected for analysis. Each bar represents the mean ± SD, n = 6. P* < 0.05.

**FIGURE 4:**
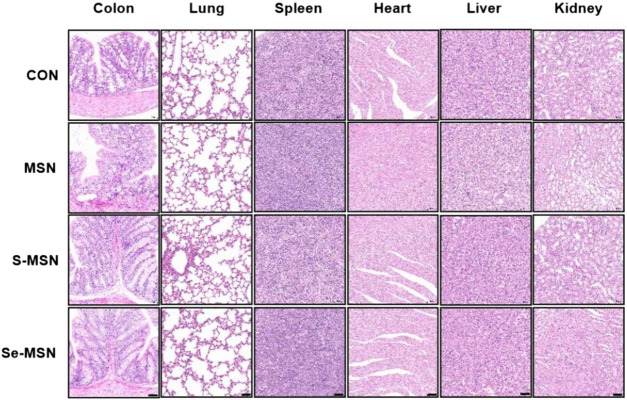
Representative H&E-stained images of major organs, including the liver, spleen, kidneys, heart, lung, and colon collected from mice after repeated challenging of three types of MSNs. Mice were treated with each MSN at 10 mg/kg daily for 1 week. Then, they were sacrificed on day 7 after the first challenging. All scale bars are 50 μm.

To assess repeated-dose toxicity, mice received daily oral doses of 10 mg/kg of each MSN type for 7 days. No significant changes in body weight, clinical signs, or mortality were observed compared to controls (Supplementary Figure S4). Repeated exposure did not result in clinical signs of toxicity, behavioural abnormalities, or material-related mortality in any group. Serum biochemical parameters—including ALB, ALP, ALT, AST, CHO, CREA, TG, TP, and UREA (Figure 2; Supplementary Table S2)—as well as hematological indices such as HGB, HCT, MCV, MCH, MCHC, and PLT (Figure 3; Supplementary Table S3), remained unchanged across all MSN groups compared to controls. Histopathological analysis of major organs at 7, 14, 28, and 90 days post-exposure revealed no treatment-related changes (Figure 4; Supplementary Figures S5–S7). These findings suggest negligible toxicity at the tested dose, although higher doses (e.g., 1.0 g/kg) have been associated with mild effects in rats. Previous studies with diselenide-bridged MSNs at 40 mg/kg for 14 days similarly showed no adverse effects, highlighting the need for further long-term, high-dose studies.

### 3.4 Biodistribution

**FIGURE 5:**
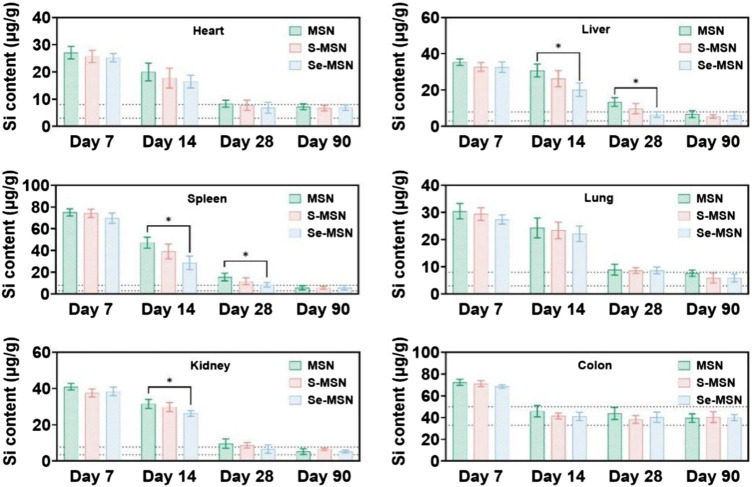
The biodistribution of three types of MSNs. Mice were treated with each MSN at 10 mg/kg daily for 1 week. Then, they were sacrificed on day 7, 14, 28 and 90 after the first challenging. Major organs, including the liver, spleen, kidneys, heart, lung, and colon were collected for quantitative measurement of Si. Each bar represents the mean ± SD, n = 6. P* < 0.05.

The biodistribution of MSNs was evaluated following repeated oral administration (10 mg/kg daily for 7 days) in mice, with silicon content in major organs (liver, spleen, kidney, lung, heart, intestine) quantified via ICP-MS. At day 1 post-exposure, all MSN variants—pure Si-O (MSN), disulfide-bridged (S-MSN), and diselenide-bridged (Se-MSN)—were detected in these organs, confirming intestinal absorption and systemic circulation (Figure 5). Plasma pharmacokinetic analysis further confirmed that all three MSN variants achieved measurable systemic exposure after oral administration. No significant differences in AUC and t_1/2_ were observed among MSN, S-MSN, and Se-MSN, indicating comparable oral absorption and blood residence profiles (Supplementary Figure S8). Silicon levels remained similar at day 7 but progressively declined, returning to baseline by day 90, indicating effective biodegradation or clearance. Notably, Se-MSN exhibited significantly lower silicon content in the liver, spleen, and kidney by day 14, and in the spleen by day 28, compared to MSN and S-MSN, suggesting accelerated clearance from the reticuloendothelial system (RES). This enhanced clearance likely stems from the rapid degradation of diselenide-bridged frameworks into soluble silicates, facilitating excretion.

Consistent with prior reports, all MSN types accumulated in the liver and spleen for at least 14 days, reflecting their retention in RES organs. The faster clearance of Se-MSN highlights the influence of framework chemistry on *in vivo* behavior. Given that silicic acid is safely absorbed and excreted, enhanced degradability is advantageous for long-term safety. Factors such as size, shape, surface charge, and morphology are known to modulate nanoparticle clearance, yet correlating these with degradability remains challenging. Furthermore, uptake by phagocytic cells (e.g., monocytes, dendritic cells) likely influences biodistribution, necessitating further studies to elucidate the interplay between MSN degradability, clearance, and toxicity.

### 3.5 Influence of MSNs on immune system

To assess the immunological safety of MSN on the immune system, we investigated their potential to induce immune dysregulation or inflammatory responses in mice. Body weight measurements across experimental groups revealed no significant differences following MSN administration compared to controls. Similarly, liver and spleen weights in MSN-treated mice were comparable to those in untreated controls, suggesting minimal immunological impact.

**FIGURE 6:**
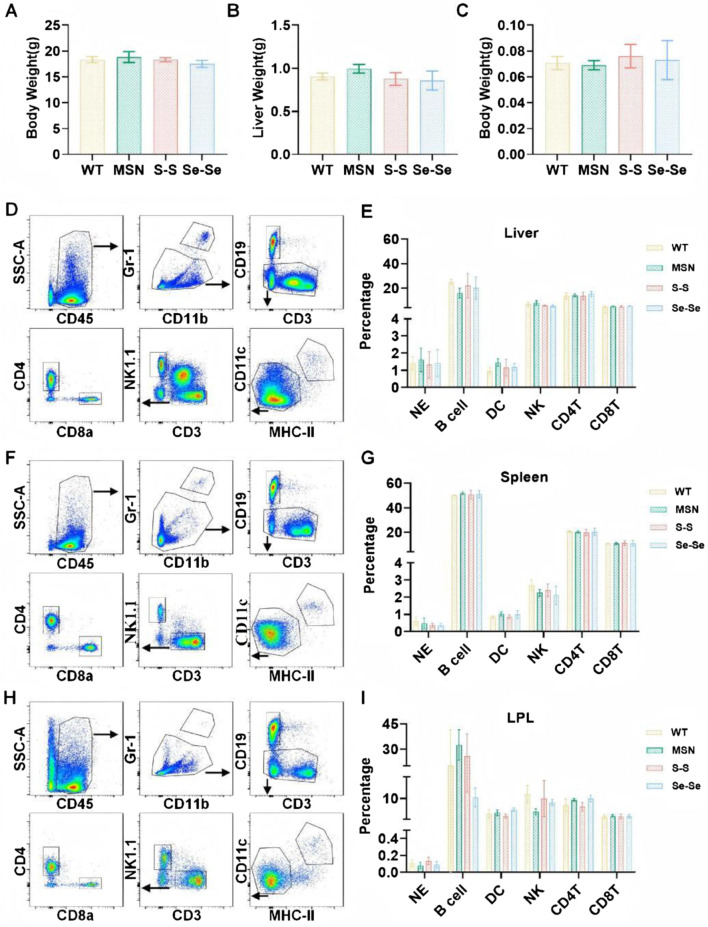
The impact of three types of MSNs on the murine immune system. Mice were treated with each MSN at a dose of 10 mg/kg/day for 1 week. **(A–C)** Body weight, liver weight, and spleen weight were measured. Immune cell populations in the liver **(D,E)**, spleen **(F,G)**, and lamina propria lymphocytes (LPL) of the small intestine were analyzed by flow cytometry **(H,I)**, including neutrophils, B cells, dendritic cells (DCs), natural killer (NK) cells, CD4+T cells, and CD8+T cells. One-way ANOVA revealed no significant differences among the groups.

For a detailed analysis, lymphocytes were isolated from the liver, spleen, and intestinal lamina propria and analyzed via flow cytometry to quantify populations of neutrophils, B cells, NK cells, CD4 T cells, and CD8 T cells (Figure 6). The immune cell composition remained consistent across all MSN-treated groups and controls, indicating that MSNs do not significantly alter immune cell profiles. Collectively, these data demonstrate that MSNs exhibit a high degree of immunological safety, with no evidence of immune dysregulation or induction of inflammatory responses, such as cytokine storms.

## 4 Conclusion

The distinct degradation profiles of S-MSN and Se-MSN, driven by their redox-sensitive disulfide and diselenide bonds, respectively, highlight the role of framework chemistry in modulating MSN behavior. The faster degradation of Se-MSN, attributed to the lower bond energy of diselenide linkages, correlates with its accelerated *in vivo* clearance, particularly from RES organs. This is a critical advancement, as prolonged nanoparticle retention can lead to toxicity concerns. The absence of toxicity across all MSN types at the tested doses suggests a favorable safety profile for oral administration. However, the mild toxicity reported at higher doses in prior studies underscores the need to explore dose-dependent effects further.

The minimal immunological impact of MSNs is particularly noteworthy, as immune activation is a key barrier to clinical translation of nanomaterials. The lack of changes in immune cell composition or inflammatory markers suggests that these MSNs are well-tolerated *in vivo*. The interplay between MSN degradability, clearance, and safety is influenced by factors such as size, surface charge, and morphology, which warrant further investigation to optimize therapeutic applications. Protein corona formation in the gastrointestinal and systemic microenvironments may also influence MSN absorption, biodistribution, and immune recognition, and should be considered in future mechanistic studies. Additionally, the uptake of MSNs by phagocytic cells (e.g., monocytes, dendritic cells) suggests that cellular interactions may modulate biodistribution, a topic requiring deeper mechanistic studies.

In summary, this study provides a comprehensive evaluation of the *in vivo* behavior of organic–inorganic hybrid MSNs with pure Si–O, disulfide-bridged, and diselenide-bridged frameworks. All MSN variants exhibited excellent biocompatibility following single and repeated oral administration in mice, with no evidence of toxicity or immune dysregulation. Diselenide-bridged MSNs demonstrated superior biodegradability and faster clearance from RES organs, offering a promising strategy for designing safer nanomaterials. To our knowledge, this is the first report of enhanced organ clearance for diselenide-bridged MSNs, marking a significant step toward tailored MSN platforms for drug delivery. Future research should focus on elucidating the mechanisms governing degradation and clearance in diverse physiological contexts and evaluating long-term safety and efficacy in complex disease models.

## Data Availability

The original contributions presented in the study are included in the article/Supplementary Material, further inquiries can be directed to the corresponding authors.
