# Supplementary material for: Toxicity of orally administrated biodegradable mesoporous silica nanoparticles in mice
Source: Front Bioeng Biotechnol. 2026 Jun 30;14:1834001. doi: 10.3389/fbioe.2026.1834001 (PMC13365342; doi:10.3389/fbioe.2026.1834001)
Supplement: Supplementary file 1 [file DataSheet1.docx]

Supplementary Material

# Supporting Information

**Toxicity of orally administrated biodegradable mesoporous silica nanoparticles in mice**

**Jie Li ^1,†^, Xin Zhao ^2,†^, Zhen Wang ^3,†^, Xiaochun Xie ^2^, Wanfen Li ^4^, Yingshi Han ^4^, Xin Tang ^5^, Jiayi Wang ^6^, Yige Shan ^6^, Fan Zhang ^7,*^, Yingshuai Wang ^8,*^, and Yi He ^4,*^**

^1^Department of Neurology, The Third Affiliated Hospital of Southern Medical University, Guangzhou, Guangdong 510630, China

^2^School of Medicine, South China University of Technology, Guangzhou, Guangdong 510006, China

^3^Department of Breast Surgery, Second Hospital of jilin University, 4026 Yatai Street, changchun 130041, china

^4^Department of Rheumatology and Immunology, The Third Affiliated Hospital of Southern Medical University, Guangzhou, Guangdong 510630, China

^5^The First Affiliated Hospital, Biomedical Translational Research Institute and School of Pharmacy, Jinan University, Guangzhou 510632, China

^6^School of Pharmacy, Xuzhou Medical University, Xuzhou 221004, China

^7^CAS Key Laboratory of Bio Medical Diagnostics, Suzhou Institute of Biomedical Engineering and Technology, Chinese Academy of Sciences, Suzhou 215163, China

^8^Department of Bioscience and Technology, Shandong Second Medical University,Weifang, Shandong 261000, China

***Correspondence:**

Fan Zhang zhangfan@sibet.ac.cn; Yingshuai Wang yingshuaiwang1987@163.com; Yi He heyi1983@smu.edu.cn

† These authors contributed equally to this work.

# Materials and Methods

**Animals and Ethics**

All animal experiments were conducted in accordance with protocols approved by the Ethics Committee for the Use of Experimental Animals at South China University of Technology. Female C57BL/6 mice, aged 6 weeks, were procured from Hunan SIA Laboratory Animal Co., Ltd. The mice were maintained on a sterilized commercial pelleted diet and provided with water ad libitum. They were housed individually in sterilized cages under controlled environmental conditions, including a temperature of 22 ± 3 °C, humidity of 55 ± 15%, and a 12-hour light/dark cycle, with virgin hardwood chips used as bedding.

**Hematological and blood biochemical assay**

Blood samples were collected via retro-orbital puncture for comprehensive hematological and biochemical analyses to assess potential toxic effects of MSNs on the treated mice. Hematological parameters, including white blood cell (WBC) count, red blood cell (RBC) count, hemoglobin (HGB) level, mean corpuscular volume (MCV), mean corpuscular hemoglobin (MCH), mean corpuscular hemoglobin concentration (MCHC), and platelet (PLT) count, were evaluated. Concurrently, biochemical markers such as alanine aminotransferase (ALT), aspartate aminotransferase (AST), alkaline phosphatase (ALP), total bilirubin (TB), albumin (ALB), blood urea nitrogen (BUN), serum creatinine (CRE), total cholesterol (TC), and total triglycerides (TG) were measured to provide a comprehensive assessment of organ function and metabolic status.

**Acute and repeated-dose oral toxicity evaluation**

For the acute toxicity study, young adult male and nulliparous, non-pregnant female C57BL/6 mice were randomly assigned to control and treatment groups. After 4 h fasting with free access to water, MSNs were administered by oral gavage at 1000 or 2000 mg/kg. Mortality and clinical signs were monitored twice daily for 14 days. At day 14, mice receiving 2000 mg/kg were euthanized, and major organs were collected for H&E-based histopathological analysis.

For the evaluation of repeated-dose toxicity, female C57BL/6 mice were randomly allocated to receive either MSNs (10 mg/kg, intragastric) or vehicle control once daily for one week. Throughout the dosing period, animals were monitored weekly for changes in appearance, behavior, and body weight. At 7, 14, 28, and 90 days following the initial administration, six mice from each group were euthanized for toxicological analysis. Major organs—including the liver, spleen, kidney, lung, heart, and colon—were collected, fixed in 10% neutral-buffered formalin, embedded in paraffin, sectioned, and stained with hematoxylin and eosin (H&E). Histopathological evaluation was performed using a digital microscope.

The acute study used 1000 and 2000 mg/kg as high-dose limit levels to assess single-dose toxicity, whereas 10 mg/kg/day was selected as a repeated working dose to evaluate biodistribution, clearance, and tissue responses under repeated exposure. This repeated-dose study was not intended to determine the maximum tolerated dose or NOAEL.

**Statistical methods**

All experimental data are presented as mean ± standard deviation. Statistical analyses were performed using GraphPad Prism 8.0 software. For comparisons among multiple groups, one-way analysis of variance (ANOVA) was employed, while factorial design ANOVA was used for intergroup comparisons. For pairwise comparisons, the least significant difference (LSD) t-test was applied when variances were homogeneous, and Dunnett’s test was used when variances were unequal. A p-value of less than 0.05 was considered statistically significant.

# Supplementary Figures and Tables


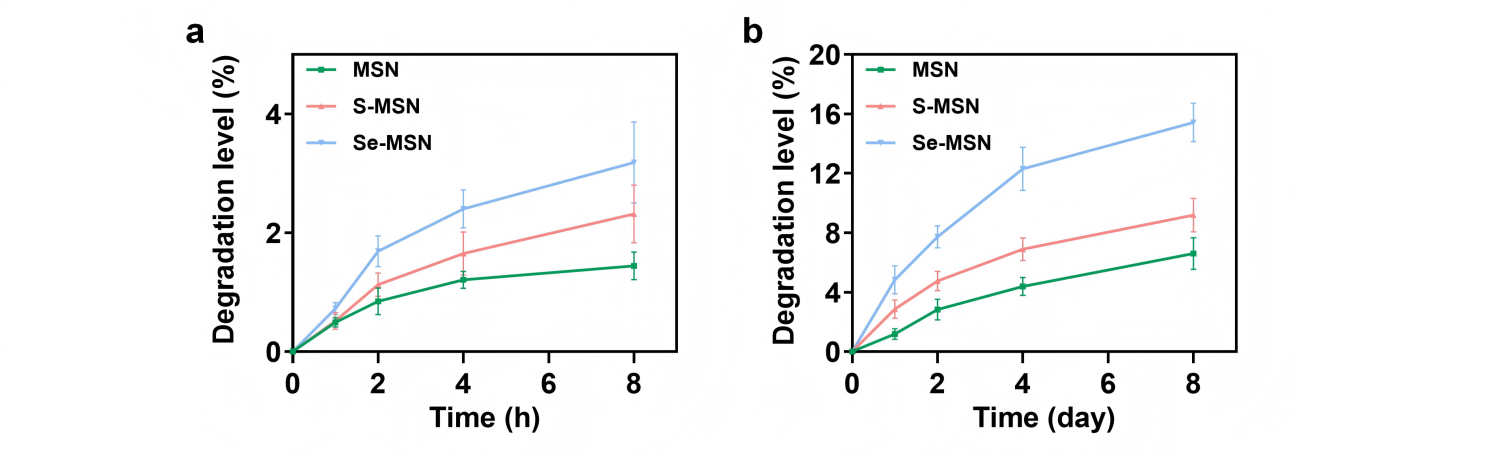


**Figure S1.** Degradation level of each MSN was determined by the quantitative measurement of Si under (**a**) simulated gastric fluid (SGF) or (**b**) simulated intestinal fluid (SIF) (n=3).


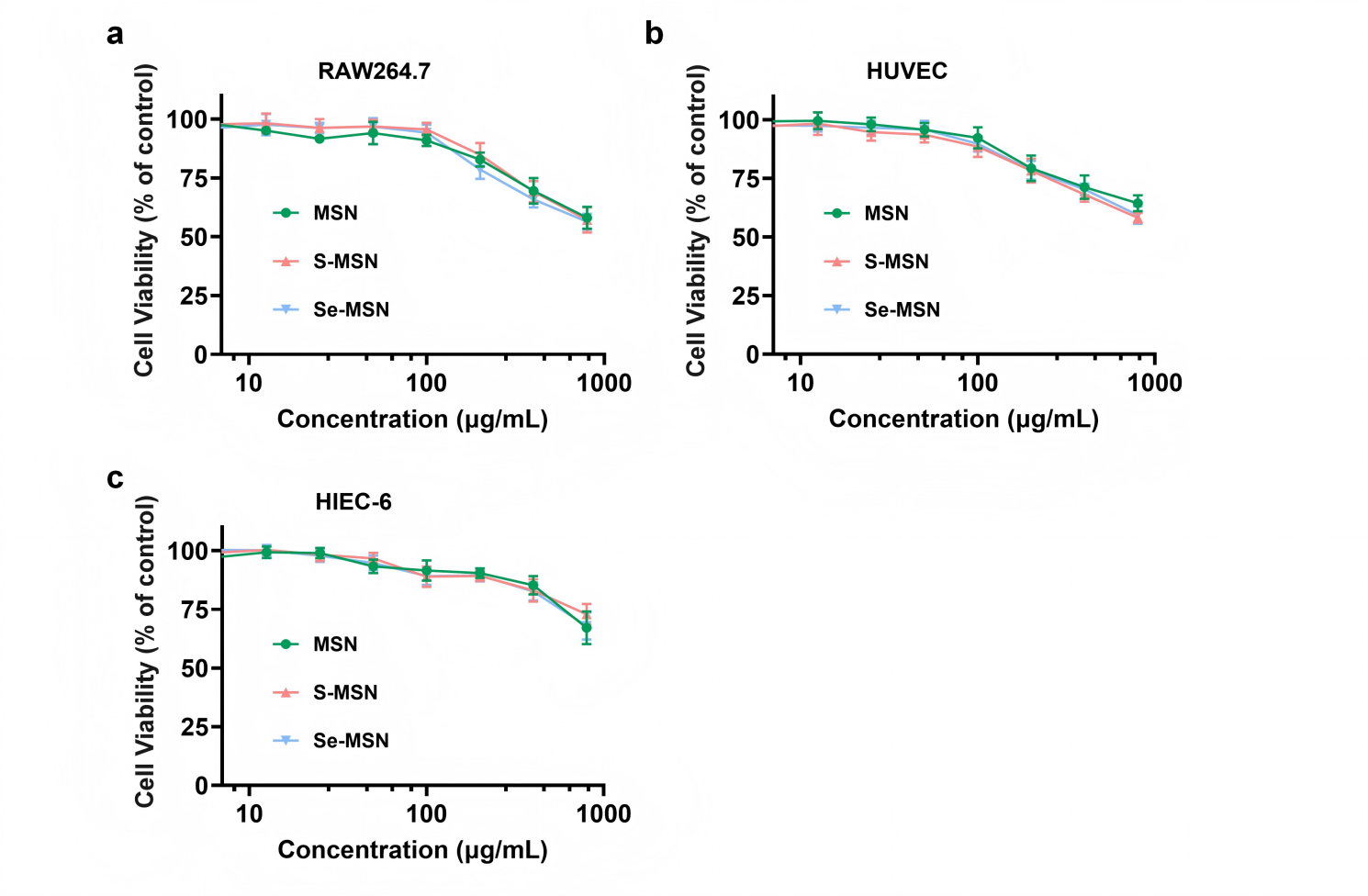


**Figure S2.** The viability of (**a)** RAW264.7 cells, (**b)** HUVEC cells and (**c)** HIEC-6 cells after incubation with MSN, S-MSN and Se-MSN for 24 h.


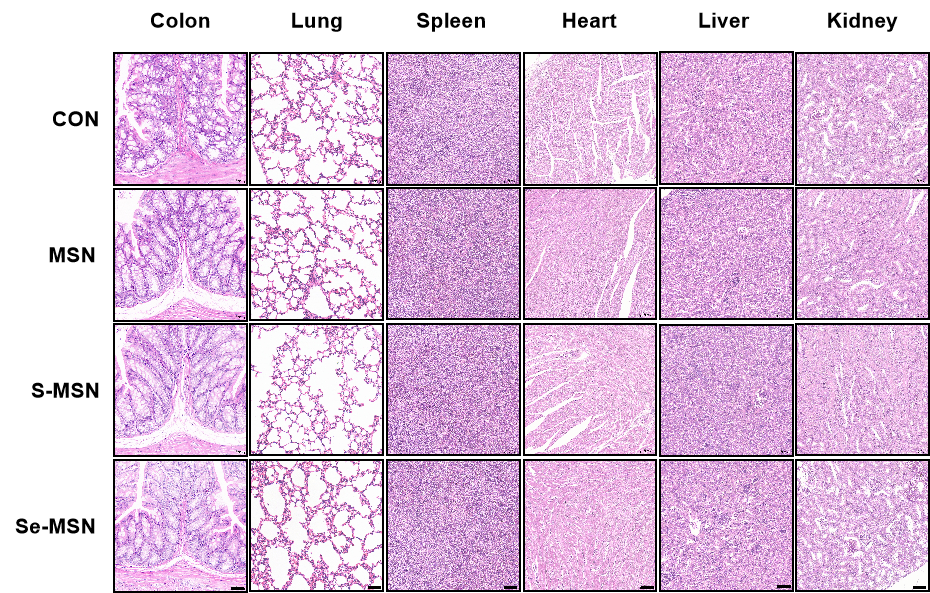


**Figure S3.** Representative H&E-stained images of major organs, including the liver, spleen, kidneys, heart, lung, and colon collected from mice at the end of treatment in different treatment groups.All scale bars are 50 μm.

**Figure S4.** Body weight changes of mice during the entire treatment period in different treatment groups (n = 6).


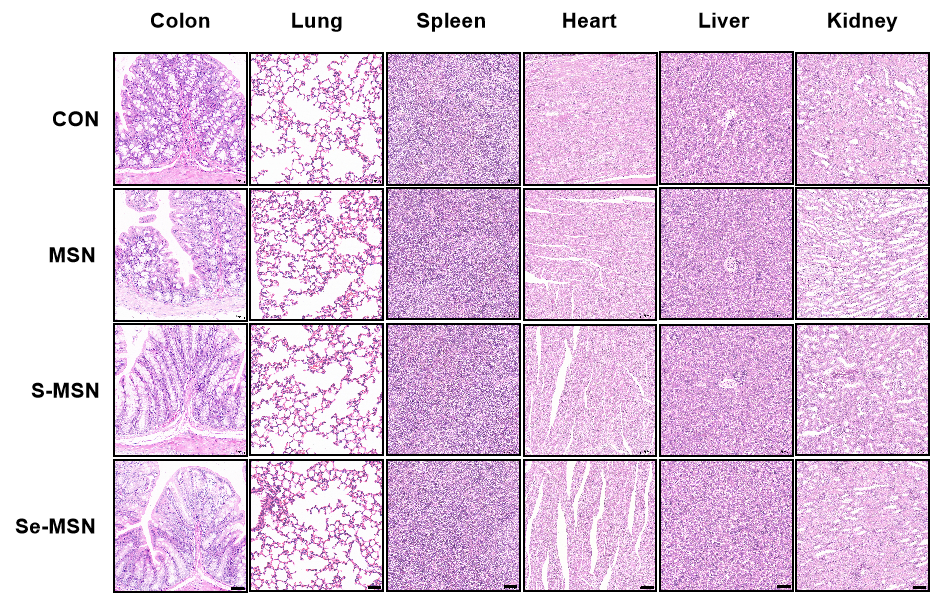


**Figure S5.** Representative H&E-stained images of major organs, including the liver, spleen, kidneys, heart, lung, and colon collected from mice after repeated challenging of three types of MSNs. Mice were treated with each MSN at 10 mg/kg daily for one week. Then, they were sacrificed on day 14 after the first challenging. All scale bars are 50 μm.


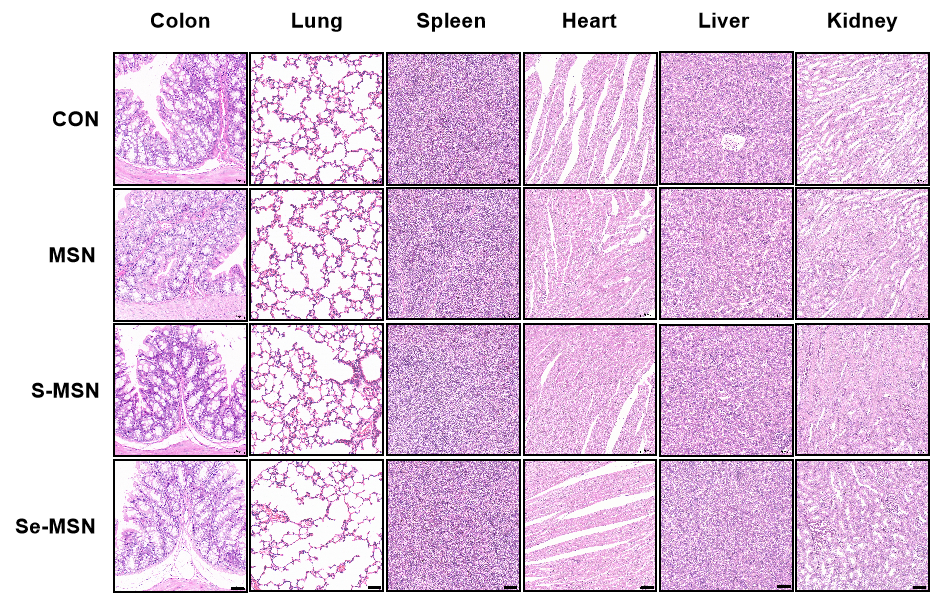


**Figure S6.** Representative H&E-stained images of major organs, including the liver, spleen, kidneys, heart, lung, and colon collected from mice after repeated challenging of three types of MSNs. Mice were treated with each MSN at 10 mg/kg daily for one week. Then, they were sacrificed on day 28 after the first challenging. All scale bars are 50 μm.


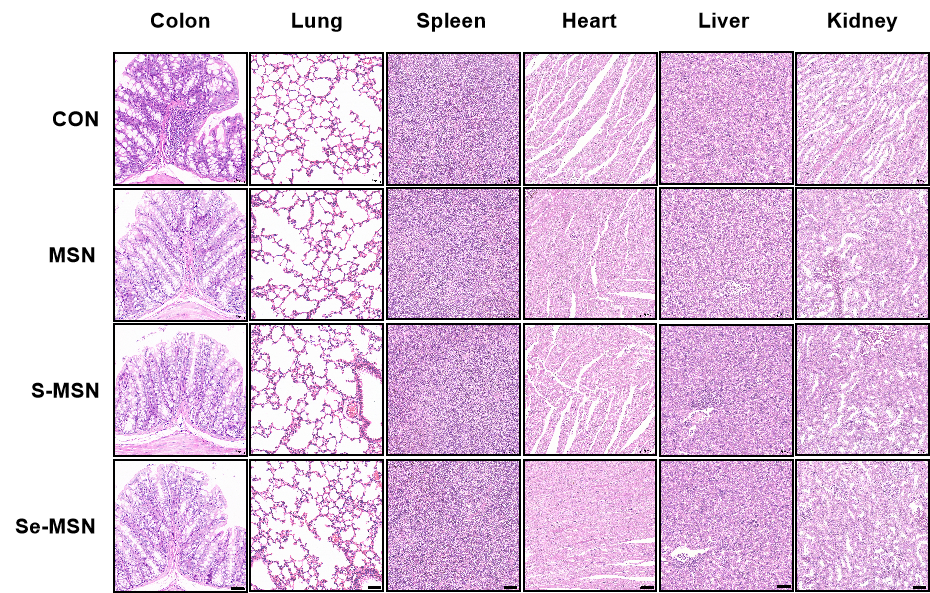


**Figure S7.** Representative H&E-stained images of major organs, including the liver, spleen, kidneys, heart, lung, and colon collected from mice after repeated challenging of three types of MSNs. Mice were treated with each MSN at 10 mg/kg daily for one week. Then, they were sacrificed on day 90 after the first challenging. All scale bars are 50 μm.


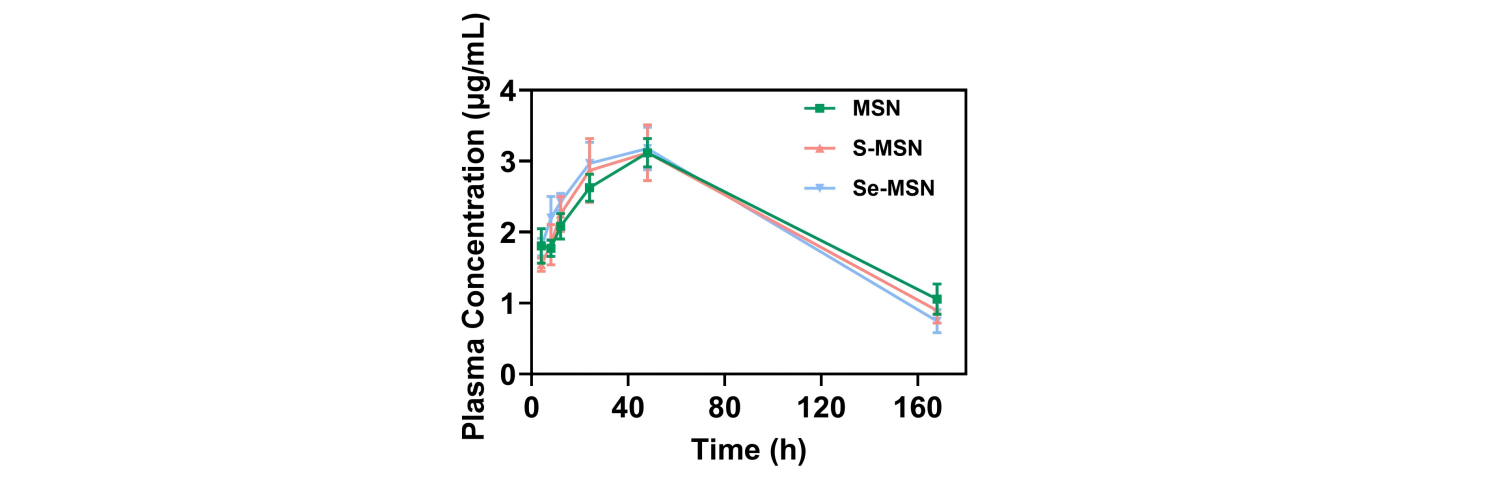


**Figure S8.**  Time-dependent plasma concentration of Si element after oral administration of three types of MSNs (n = 3).

**Table S1.** Physicochemical characterization of three types of MSNs.

|  | Particle size   (nm) | Zeta potential   (mV) | BET  (m2 g−1) | Pore size   (nm) | Pore volume  (cm3 g−1) |
| --- | --- | --- | --- | --- | --- |
| MSN | 108.9±10.3 | -23±2.1 | 523.3 | 5.8 | 1.24 |
| S-MSN | 119.4±8.5 | -20±3.5 | 492.6 | 6.2 | 1.19 |
| Se-MSN | 112.3±12.3 | -24±4.3 | 55.8 | 6.5 | 1.06 |

**Table S2**. The blood biochemistry data of mice on day 7, 14, 28 and 90 after repeated challenging of three types of MSNs. (Mean ± SD)

|  | Group | ALB(g/L) | ALP(U/L) | ALT(U/L) | AST(U/L) | TC (mmol/L) | CREA (μmol/L) | TG (mmol/L) | TP (mmol/L) | UREA (mmol/L) |
| --- | --- | --- | --- | --- | --- | --- | --- | --- | --- | --- |
| Day1 | CON | 59.17±2.6 | 132.50±13.35 | 136.08±9.12 | 39.92±1.59 | 110.67±8.16 | 3.07±0.43 | 24.63±2.06 | 2.76±0.38 | 54.42±0.84 |
|  | MSN | 57.83±3.33 | 137.08±9.47 | 137.25±12.12 | 34.50±2.38 | 110.67±3.47 | 3.60±0.15 | 25.72±4.19 | 3.54±0.5 | 54.87±0.38 |
|  | S-MSN | 56.33±4.69 | 135.42±16.98 | 131.33±7.68 | 38.5±4.54 | 104.17±9.39 | 2.73±0.37 | 24.23±3.51 | 2.76±0.2 | 55.20±0.19 |
|  | Se-MSN | 62.17±3.01 | 157.67±5.14 | 147.50±9.02 | 37.50±5.38 | 115.08±4.77 | 2.63±0.45 | 24.90±0.18 | 2.87±0.84 | 55.53±0.19 |
| Day7 | CON | 58.08±3.61 | 139.25±8 | 142.25±7.56 | 38.67±4.23 | 100.58±10.06 | 3.25 ±0.09 | 23.63±3.36 | 2.76±0.2 | 54.42±0.38 |
|  | MSN | 54.92±1.66 | 134.00±9.81 | 137.75±10.5 | 35.75±4.27 | 103.50±9.54 | 3.12±0.45 | 24.75±3.33 | 2.87±0.51 | 54.31±0.57 |
|  | S-MSN | 59.00±4.12 | 146.75±12.88 | 147.00±12.76 | 35.42±4.49 | 98.75±12.79 | 2.92±0.66 | 28.47±0.68 | 2.77±0.69 | 54.87±0.51 |
|  | Se-MSN | 57.33±5.34 | 134.42±9.27 | 144.50±13.76 | 34.08±2.88 | 106.42±13.37 | 3.27±0.84 | 25.72±2.69 | 3.20±0.39 | 55.64±0.01 |
| Day14 | CON | 58.42±4.16 | 148.08±6.98 | 137.75±7.57 | 37.33±2.04 | 101.00±2.82 | 2.50±0.09 | 27.63±2.07 | 2.43±0.5 | 54.42±0.2 |
|  | MSN | 58.58±1.23 | 130.92±4.51 | 135.17±13.80 | 34.67±1.53 | 107.83±2.43 | 2.53±0.66 | 25.33±3.43 | 3.21±0.69 | 54.76±0.19 |
|  | S-MSN | 57.58±1.66 | 146.58±16.96 | 133.00±10.07 | 34.00±1.39 | 112.58±6.57 | 2.35±0.23 | 25.90±3.48 | 3.32±0.34 | 54.98±0.67 |
|  | Se-MSN | 61.75±2.18 | 133.17±6.93 | 142.50±16.14 | 38.25±2.05 | 117.25±1.3 | 2.40±0.32 | 24.84±1.18 | 3.10±0.51 | 53.98±0.57 |
| Day28 | CON | 59.58±2.57 | 147.42±12.51 | 142.58±17.14 | 36.00±3.38 | 107.67±15.13 | 3.07±0.66 | 24.29±2.09 | 3.10±0.19 | 54.98±0.88 |
|  | MSN | 61.58±2.13 | 140.83±6.66 | 148.33±3.39 | 39.33±4.19 | 110.92±12.87 | 2.78±0.65 | 27.17±1.25 | 3.54±0.51 | 54.76±0.19 |
|  | S-MSN | 63.83±1.66 | 141.58±4.59 | 147.17±8.27 | 35.67±4.86 | 93.33±4.71 | 3.60 ±0.26 | 23.93±2.96 | 2.88±0.51 | 54.20±0.57 |
|  | Se-MSN | 57.33±5.2 | 130.75±8.84 | 139.75±15.38 | 35.75±4.21 | 103.25±11.05 | 2.68±0.66 | 23.63±2.35 | 2.43±0.51 | 54.97±0.58 |
| Day90 | CON | 59.58±3.73 | 129.08±8.84 | 133.58±13.84 | 37.92±2.52 | 107.17±10.59 | 3.35±0.17 | 26.96±2.46 | 3.21±0.20 | 55.42±0.19 |
|  | MSN | 61.58±3.92 | 140.17±11.82 | 142.67±12.05 | 34.83±1.13 | 113.92±1.38 | 3.45±0.15 | 24.51±3.88 | 2.98±0.01 | 54.87±0.38 |
|  | S-MSN | 63.83±1.94 | 149.25±12.99 | 134.33±9.23 | 37.42±2.6 | 105.00±10.64 | 2.73±0.58 | 26.78±2.59 | 2.77±0.39 | 54.54±0.51 |
|  | Se-MSN | 57.33±4.91 | 132.33±6.01 | 135.00±17.61 | 38.67±4.26 | 103.42±13.56 | 3.07±0.58 | 25.08±2.91 | 3.32±0.88 | 55.31±0.58 |

**Table S3.** The hematological factors of mice on day 7, 14, 28 and 90 after repeated challenging of three types of MSNs. (Mean ± SD)

|  | Group | WBC (10^9/L) | RBC (10^12/L) | HGB (g/L) | HCT (%) | MCV (fL) | MCH  (pg) | MCHC (g/L) | PLT (10^9/L) |
| --- | --- | --- | --- | --- | --- | --- | --- | --- | --- |
| Day7 | CON | 4.49±0.9 | 9.06±0.91 | 144±7 | 45.30±1.47 | 48.07±0.61 | 15.50±0.45 | 32 ±6 | 871±55 |
|  | MSN | 4.22±0.5 | 9.57±0.19 | 146±4 | 45.37±0.49 | 47.43±0.39 | 15.23±0.33 | 32±7 | 958±87 |
|  | S-MSN | 4.41±0.51 | 10.19±0.17 | 155±1 | 48.17±0.41 | 47.27±0.41 | 15.23±0.12 | 323±1 | 1002±116 |
|  | Se-MSN | 4.14±0.52 | 9.81±0.22 | 152±4 | 47.30±1.61 | 48.20±0.64 | 15.47±0.12 | 321±5 | 791±147 |
| Day14 | CON | 3.95±0.32 | 9.58±0.83 | 144±5 | 43.43±4.11 | 45.60±0.92 | 14.83±0.52 | 322±4 | 912±96 |
|  | MSN | 5.10±0.66 | 9.95±1.38 | 149±6 | 42.93±1.52 | 46.32±2.5 | 15.71±0.16 | 316±3 | 96±26 |
|  | S-MSN | 3.82±0.21 | 10.01±0.2 | 153±4 | 44.54±0.87 | 45.87±2.79 | 14.93±0.13 | 321±3 | 949±77 |
|  | Se-MSN | 4.17±0.4 | 9.29±0.83 | 149±4 | 44.17±2.01 | 49.20±1.68 | 14.96±0.51 | 317±4 | 921±43 |
| Day28 | CON | 5.17±0.97 | 10.23±1.13 | 155±9 | 45.91±2.98 | 44.58±1.98 | 16.0 ±0.4 | 313±3 | 900±26 |
|  | MSN | 4.81±0.56 | 9.63±1.67 | 152±9 | 46.03±3.66 | 45.66±1.84 | 15.58±0.36 | 324±0 | 907±51 |
|  | S-MSN | 4.36±0.57 | 10.65±0.03 | 14 3±8 | 42.45±0.92 | 44.85±1.78 | 15.26±0.05 | 325±5 | 881±48 |
|  | Se-MSN | 4.40±0.9 | 12.67±4.26 | 147±10 | 46.03±2.62 | 48.0±2.29 | 15.80±0.17 | 323±3 | 860±9 |
| Day90 | CON | 4.36±0.78 | 9.39±0.82 | 154±22 | 45.25±1.5 | 45.6±0.55 | 15.47±0.34 | 323±2 | 884±37 |
|  | MSN | 4.00±0.83 | 9.30±0.77 | 147±4 | 46.24±1.32 | 47.39±1.13 | 15.55±0.26 | 319±2 | 912±48 |
|  | S-MSN | 4.40±0.68 | 10.71±0.28 | 146±7 | 46.15±2.58 | 49.37±1.45 | 15.56±0.31 | 318±5 | 928±64 |
|  | Se-MSN | 4.44±1.02 | 9.76±0.58 | 145±4 | 45.61±0.13 | 47.66±1.84 | 15.64±0.34 | 317±3 | 930±49 |
